# Editorial: Spotlight on aging: physiology, prevention and management of gait and balance

**DOI:** 10.3389/fphys.2024.1477957

**Published:** 2024-09-17

**Authors:** Rafael Reimann Baptista, Leonardo Alexandre Peyré-Tartaruga, Urs Granacher

**Affiliations:** ^1^ Pontifical Catholic University of Rio Grande do Sul, School of Health and Life Sciences, Porto Alegre, Brazil; ^2^ Human Locomotion Laboratory (LocoLab), Department of Public Health, Experimental Medicine and Forensic Sciences, University of Pavia, Pavia, Italy; ^3^ LaBiodin Biodynamics Laboratory, Universidade Federal do Rio Grande do Sul, Porto Alegre, Brazil; ^4^ Department of Sport and Sport Science, Exercise and Human Movement Science, University of Freiburg, Freiburg, Germany

**Keywords:** mobility, dynamic stability, older adults, exercise, dual-tasking

## Introduction

As human beings grow older, (>60 years), adequate levels of dynamic balance are needed to successfully perform activities of daily living. The ability to maintain dynamic balance directly impacts an individual’s quality of life and the risk of falls among older adults. Therefore, understanding how aging and various interventions affect balance is vital for developing strategies to enhance dynamic balance and prevent falls, contributing to healthier aging.

The multidisciplinary approach of the *Frontiers in Physiology* journal, focusing on the physiology of living systems, aligns with the need to address critical aspects of autonomy and functionality in older populations. Accordingly, this editorial synthesizes recent research findings on the effects of biological aging and interventions on dynamic balance, particularly within the context of older adults’ interaction with their environment.

The maintenance of dynamic balance and mobility constitute key components of healthy aging and are underpinned by regular physical exercise. Dynamic balance describes an individual’s ability to maintain the vertical projection of the center of mass within a small base of support (feet) during standing and walking (Horak et al., 1997). Mobility refers to the ability to move oneself within one’s living space—whether at home, in the community, or beyond—through walking, the use of assistive devices, or transportation (Rantakokko et al., 2013). Aging is a universal process characterized by a reduced capacity to adapt to environmental variations and a decrease in functionality, driven by both genetic and environmental factors (Lopez-Otín et al., 2013). Among the physiological changes associated with aging, impairments in balance and mobility are particularly significant for both the individual and society, making the study of these elements crucial.

This Research Topic aimed to deepen our understanding of various aspects of mobility, gait, dynamic balance, and dual-tasking (i.e., concurrent performance of a balance task with a secondary cognitive or motor task) in older adults. The guest editors invited submissions that explored the physiological and biomechanical foundations of these areas and their changes with biological aging. Contributions included studies examining the effects of negative aging stereotypes, as well as age-related acute adaptations on dynamic balance and cognition in both young and older adults. This Research Topic sought to offer comprehensive insights into the factors influencing mobility and dynamic balance in older adults, providing valuable information for enhancing interventions and strategies to promote healthy aging.

The Research Topic attracted significant interest, with 183 potential authors contacted by the guest editors. The Research Topic’s impact is highlighted by its total of 1,313 downloads and 6,156 views (as of September 9th, 2024), underscoring its relevance and importance within the scientific and medical communities.

## Summary of selected articles

1. Age-based stereotype threat effects on dynamic balance in healthy older adults (Borel et al.): This study explored the impact of stereotype threat on dynamic balance among older adults. The researchers investigated whether negative aging stereotypes affected balance performance and sought methods to counteract these effects. Twenty-two participants aged 65–75 years were tested under conditions of stereotype threat and reduced threat. With negative aging stereotypes, the authors referred to a phenomenon where negative beliefs about aging reduce older adults’ cognitive performance. Participants performed balance tasks on a moving platform under varying difficulty levels (e.g., with open or closed eyes on firm or foam surfaces). Findings showed that participants under stereotype threat exhibited poorer balance, particularly in more demanding conditions, with less effective stabilization of body segments. Conversely, those in the reduced threat condition maintained a better balance. These findings suggest that aging stereotype threat can negatively affect balance by diverting attention. Adequate interventions may mitigate negative aging stereotypes by improving balance in older adults.
2. Natural ageing primarily affects the initial response to a sustained walking perturbation but not the ability to adapt over time (Swart et al.): The study examined how natural aging affects the ability to respond to and adapt to sustained gait perturbations. Using a split-belt treadmill paradigm, the study included 75 healthy adults aged 18–79 years. Participants walked on the treadmill with different belt speeds, simulating a gait perturbation. The authors found that the flexibility to respond to unexpected gait perturbations decreased with age, evidenced by greater initial gait asymmetry. However, the short-term ability to adapt over time to these perturbations was not affected by age. Of note, older adults required more steps to reach a stable adaptation level. These study findings suggest that while aging reduces the initial flexibility in gait responses, the capacity for acute adaptation remains intact, highlighting the need for targeted interventions to enhance initial gait responses in older populations.
3. The effect of mobile phone task and age on gait (Zhang et al.): The authors of this meta-analysis investigated how the usage of mobile phones affects gait parameters in individuals aged 18 to 61 years. The study synthesized data from 22 studies with 592 participants who performed dual-tasks using a mobile phone while walking. The type of dual-tasks included texting, reading, dialing, and talking while walking. The analysis revealed that attentionally demanding tasks such as texting and reading significantly decreased gait velocity, stride length, and step length, while increasing step time, stride time, step width, and double support time. In contrast to young adults, mobile phone use did not affect step length and step time in older participants.
4. Unraveling age-related impairment of the neuromuscular system: exploring biomechanical and neurophysiological perspectives (Nùñez-Lisboa et al.): The main aim of this narrative review was to describe the age-related remodeling of both, the neural and muscular systems and how they are related to age-related locomotor changes. A secondary aim was to compared age-related gait changes in older adults with immature gait patterns of children. Finally, the authors describe effective interventions to counteract or even reverse age-related gait changes. The observed gait changes in older adults appear to emerge from a lack of propulsive lower limb muscle power due to age-related motor unit remodeling and larger proportions of slow twitch muscles fibres. In addition, this narrative review outlines great similarities between the gait pattern of infants and that of older adults. Age-related changes in gait can be counteracted through physical exercise (e.g., resistance and power training) and novel techniques such as direct spinal stimulation or biofeedback.

Although small, this Research Topic brought together articles that fill important gaps on gait and mobility disorders due to age-related neuromuscular processes that were examined using different methodological approaches such as gait perturbations and/or dual-tasking (Swart et al.; Nùñez-Lisboa et al.; Zhang et al.) or negative aging stereotypes (Borel et al.). Findings from the four studies included highlight the need for integrative and biopsychosocial therapeutic approaches. The prescription of physical exercise therapies should be considered through an individualized analysis considering the aspects mentioned above. In addition, this Research Topic suggests specific interventions with high potential for combating movement disorders, such as direct spinal stimulation to mitigate the degeneration of spinal segments (Nùñez-Lisboa et al.), interventions with cognitive and motor dual task similar to daily activities such as texting on cell phones (Zhang et al.), interventions using controlled perturbations (Swart et al.) and approaches avoiding negative stereotypes of aging (Borel et al.).

## Conclusion

The studies presented in this Frontiers Research Topic highlight the multifaceted nature of dynamic balance in young and older adults and the impact of typical or everyday dual-task situations on gait. Moreover, counteracting negative stereotypes, such as age-fair instructions, and interventions based on biomechanical feedback may further enhance balance performance by reducing attentional distractions.

## Future research directions

To advance our understanding of dynamic balance and mobility in older adults, future research should focus on exploring the long-term effects of combined cognitive-motor interventions on balance, gait and mobility. Investigating the underlying neuromechanical mechanisms that contribute to balance and mobility improvements can help refine intervention strategies. Additionally, more research is needed to assess the effectiveness of personalized interventions tailored to individual and intrinsic fall risk situations, particularly during dual-tasking. Expanding studies to include diverse populations and environments will provide a more comprehensive understanding of how different factors influence dynamic balance and fall risk in aging populations. Finally, integrating advanced technologies like wearable sensors and machine learning algorithms could offer real-time feedback and more precise assessments, further enhancing the efficacy of interventions aimed at promoting dynamic balance and mobility by preventing falls and maintaining older adults’ quality of life.

## References

[B1] Horak F. B. Henry S. M. Shumway-Cook A. Postural perturbations: new insights for treatment of balance disorders. Phys. Ther. 1997;77(5):517–533. 10.1093/ptj/77.5.517 9149762

[B2] Lopez-Otín C. Blasco M. A. Partridge L. Serrano M. Kroemer G. The hallmarks of aging. Cell. 2013;153(6):1194–1217. 10.1016/j.cell.2013.05.039 23746838 PMC3836174

[B3] Rantakokko M. Mänty M. Rantanen T. Mobility decline in old age. Age Ageing. 2013;42(5):521–527. 10.1093/ageing/aft058 23038241

